# A combination network of CNN and transformer for interference identification

**DOI:** 10.3389/fncom.2023.1309694

**Published:** 2023-12-06

**Authors:** Hu Zhang, Meng Zhao, Min Zhang, Sheng Lin, Youqiang Dong, Hai Wang

**Affiliations:** School of Aerospace Science and Technology, Xidian University, Xi'an, China

**Keywords:** communication interference identification, electronic countermeasures, convolutional neural network, transformer, cross-attention mechanism

## Abstract

Communication interference identification is critical in electronic countermeasures. However, existed methods based on deep learning, such as convolutional neural networks (CNNs) and transformer, seldom take both local characteristics and global feature information of the signal into account. Motivated by the local convolution property of CNNs and the attention mechanism of transformer, we designed a novel network that combines both architectures, which make better use of both local and global characteristics of the signals. Additionally, recognizing the challenge of distinguishing contextual semantics within the one-dimensional signal data used in this study, we advocate the use of CNNs in place of word embedding, aligning more closely with the intrinsic features of the signal data. Furthermore, to capture the time-frequency characteristics of the signals, we integrate the proposed network with a cross-attention mechanism, facilitating the fusion of temporal and spectral domain feature information through multiple cross-attention computational layers. This innovation obviates the need for specialized time-frequency analysis. Experimental results demonstrate that our approach significantly improves recognition accuracy compared to existing methods, highlighting its efficacy in addressing the challenge of communication interference identification in electronic warfare.

## 1 Introduction

Interference identification has received increasing attention in military and civilian applications (Zhang et al., 2013). Interference identification aims at recognizing the category of interference without any prior information, which is of great importance for anti-interference communications.

Interference identification methods are commonly classified into two categories: feature-based and learning-based methods. Feature-based techniques utilize parameters such as amplitude, phase, and wavelet transform as extracted features for classifiers (Ibrahim et al., 2019; Nishio et al., 2019). In their work, Zhang and Cao (2018) introduced a waveform classification approach based on Support Vector Machines (SVM) tailored for automotive radar interference.

Subsequently, the integration of machine learning and swarm intelligence techniques has shown significant promise in yielding exemplary outcomes across diverse fields (Malakar et al., 2020; Bacanin et al., 2021, 2022; Ramanan et al., 2022; Tuba et al., 2022).

Recently, the widespread adoption of deep learning has garnered significant attention in various fields, including the analysis of clustered weather patterns (Chattopadhyay et al., 2020), as well as image detection (Qian et al., 2021; Lin et al., 2022; Zhou et al., 2022; Lin et al., 2023) and processing (Gawande et al., 2022; Zivkovic et al., 2022; Zhang et al., 2023). Benefitting from the powerful feature extraction capability of deep learning, learning-based methods also have achieved good performance in identification of communication signals (Kattenborn et al., 2021; Sun et al., 2021). O’Shea et al. (2016) used convolutional neural networks (CNNs) to classify wireless modulated signals, and the effectiveness of the method was experimentally demonstrated. After that, Schmidt et al. (2021) used CNNs to study the automatic recognition of interference signals. Due to the simple structure of the network, the recognition accuracy could also be improved.

In Li et al. (2019), carried out radio signal recognition method based on gated recurrent unit (GRU). Compared to CNNs, GRU has more advantages in feature extraction of one-dimensional signals. However, it is difficult to make GRU into a multi-layer structure and that limits its feature extraction capability for long sequences. Residual network (ResNet) was employed for modulation mode identification in West and O’Shea (2017). The method alleviates the problem of gradient decay in deeper networks. However, excessive use of the residual structure can also lead to a larger amount of model parameters and waste of computational resources. In Zhang et al. (2018), a combination of CNNs and long short-term memory (LSTM) was proposed and experimental results showed that it has better recognition performance than either CNNs or LSTM. It was shown that the effective combination of composite networks can improve recognition results. Zhang et al. (2019) constructed four classical neural network models to identify three types of wireless interference signals, which demonstrate the generality of the effectiveness of deep learning at the considered task. Wang et al. (2020) achieved satisfactory results in modulation mode classification by using two CNNs for weight sharing and designing a new loss function. Influenced by the development of transformer (Vaswani et al., 2017; Dosovitskiy et al., 2021; Liu et al., 2021), the utilizations of transformer in signal recognition field (Huang et al., 2022; Wang et al., 2022a) have achieved better performance than CNNs. In Wang et al. (2022b), short-time Fourier transform (STFT) was used for time-frequency analysis, and this method exploits the multi-domain information of the signal. However, signals in different domains need to be processed with different branched networks, while the dedicated time-frequency analysis step adds to the process of interference identification.

Inspired by the above study, we explore the application of transformer in interference identification. Moreover, considering that the disadvantage of transformer in local feature capture capability, this paper designs a novel network architecture, which combines CNNs and transformer (CNNTF). This fusion is not only unique, but also enables more comprehensive signal analysis. In summary, this paper makes the following contributions:

- Firstly, we introduce a CNNTF network. In contrast to the conventional practice of employing simple network combinations, this paper introduces a novel approach by utilizing CNNs in lieu of word embedding. This decision stems from the recognition of the inherent complexity associated with contextual semantics in signal data, which poses challenges for comprehension using word embedding techniques. This modification significantly enhances the network’s applicability in extracting features from signal data, which equip it with both local and global extraction capabilities.
- In addition, we integrated CNNTF with a cross-attention mechanism (CNNTF-CA) to exploit the correlations between different features. This integration allows the network to extract multiple domain features simultaneously, without requiring any special time-frequency analysis. As a result, the network can associate time-domain and frequency-domain features effectively. Our approach represents an innovative way to enhance the capabilities of neural networks for feature extraction.
- The experimental results validate the effectiveness of the proposed method.

## 2 Signal model

In this section, five types of single interference signals, which consists of single-tone (ST), multi-tone (MT), linear sweep (LS), partial band noise (PBN) and noise frequency modulation (NFM), are used. The signal model can be denoted as


(1) $$R\left(t\right)=S\left(t\right)e^{j\left(2\pi f_{s}t+\varphi_{s}\left(t\right)\right)}+J\left(t\right)e^{j\left(2\pi f_{J}t+\varphi_{J}\left(t\right)\right)}+W\left(t\right)$$

where $R\left(t\right)$ represents the received signal. $S\left(t\right)$ is communication signal, $f_{s}$ and $\varphi_{s}\left(t\right)$ are separately carrier frequency and initial phase of $S\left(t\right)$ . $J\left(t\right)$ is jamming signal, $f_{J}\left(t\right)$ and $\varphi_{J}\left(t\right)$ are carrier frequency and initial phase of $J\left(t\right)$ , respectively. $W\left(t\right)$ is additive white Gaussian noise (AWGN).

Additionally, the interference signals can be expressed in both time-domain and frequency-domain. Frequency domain data can be obtained from time domain data by fast Fourier transform (FFT), which can be written as


(2) $$x\left(k\right)=\overset{N-1}{\underset{n=0}{\sum }}x\left(n\right)e^{-j2\pi kn/N}$$

where $e^{-j2\pi /N}$ denotes the rotation factor. n and k denote the discrete points in the time and frequency domains, respectively. j is the imaginary part.

After that, take the amplitude and phase of the FFT data to obtain the amplitude spectrum and phase spectrum data.

## 3 Methods

In this paper, we propose a CNNTF method which combines CNNs and transformer. Based on CNNTF, we introduce a cross-attention mechanism to design the CNNTF-CA model, which can effectively fuse features from different domains to achieve the purpose of time-frequency analysis.

### 3.1 CNNTF

The CNNTF is designed to combine CNN and the encoding of transformer, discarding the word embedding layer of transformer. The utilization of this module has two main advantages. Firstly, for communication interference signals, the local correlation between adjacent sampling points affects the training effect of the model and should not be ignored. CNNs has the advantage of local connectivity in learning features specifically for features between adjacent samples of the signal sequence. Secondly, considering the complexity in extracting contextual semantics from 1D signal data, CNNs are deemed more appropriate than word coding for effectively addressing the practical challenges in this task.

The structure of the CNNs module is as follows. The dimensional convolution kernel scans the interfering data sequence first. In order to avoid gradient dissipation, batch normalization (BN) and rectified linear unit (ReLU) activation function processing are performed after the convolutional operation.

The mathematical expressions below can model the operations of the local 1-D convolution module:


(3) $$\begin{array}{l} O_{c}=F_{c}\left(I,\theta \right)\\ O_{\mathrm{ReLU}}=\mathrm{ReLU}\left(F_{BN}\left(WO_{c}\right)\right) \end{array}$$

where $F_{c}\left(\cdot \right)$ means the convolution function, I is the input signal and θ is the parameter in CNNs. $F_{BN}$ denotes the BN processing, and W stands for the weight of convolutional layer. In addition, $O_{c}$ and $O_{\mathrm{ReLU}}$ are the output of CNNs layer and the ReLU activation layer, respectively.

The transformer module consists of an attention layer (AL) and a feedforward network (FFN). The attention function can be described as


(4) $$\mathrm{Attention}\left(Q,K,V\right)=\mathrm{softmax}\left(\frac{QK^{T}}{\sqrt{d}}\right)V$$

where Q,K and V are the query, key and value matrices separately.

The FFN is used after AL, which is composed of two linear translation layers. After the first linear layer, a ReLU activation function is employed, and the whole process can be described as


(5) $$FFN\left(x\right)=\mathrm{ReLU}\left(W_{2}\left(W_{1}x+b_{1}\right)\right)+b_{2}$$

where $W_{1}\in R^{C\times C}$ and $W_{2}\in R^{C\times C}$ can be used to describe the weights of different layers, separately; $b_{1}$ and $b_{2}$ denote the offset quantity of different layers, respectively.

There is an interlayer between the attention and FFN layers, which consists of residual connection (RC) and layer normalization (LN). The reason for using the residual connection is to prevent gradient dissipation with the network depth increasing, which can be formulated as follows:


(6) $$x_{l+1}=H\left(x_{l}\right)+F\left(x_{l},W_{l}\right)$$

where $x_{l}$ and $x_{l+1}$ are the input and output vectors of the lth layer, respectively. $H\left(x_{l}\right)$ means the direct mapping; $F\left(x_{l},W_{l}\right)$ represents the residual mapping. All the layers use residual connections to each other. LN follows RC in the interlayer, which provides better performance for the processing of batches with small size.

To ensure that the dimension of the output is consistent with that of the previous layer, a one-dimensional deconvolution layer is needed to reduce the dimension before the output. Then, after the linear layer and normalization, realized by SoftMax function, the output result is obtained.

### 3.2 Cross-attention mechanism

**Figure 1:**
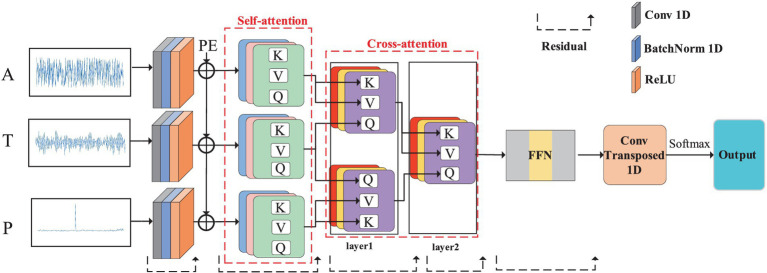
The structure diagram of CNNTF-*CA.* The CNNTF-CA contains structure of CNNTF, and PE is the positional encoding. A, T, and P represent amplitude spectrum, time domain and phase spectrum data.

The time domain and frequency domain are the basic properties of the communication signals. In the field of signal processing, there are usually special time-frequency analysis steps to combine the time-frequency domain data, which will also make the interference identification process more complex. Therefore, this paper introduces the cross-attention mechanism to combine the characteristics of time domain and frequency domain to play the role of time-frequency analysis. In this paper, in order to reduce the time-frequency analysis process, a cross-attention mechanism is used to correlate the data from two different domains. The overall structure of CNNTF-CA is shown in Figure 1.

**Figure 2:**
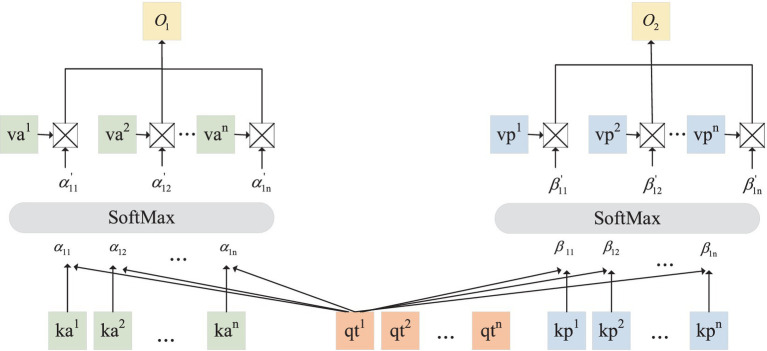
Cross-attention calculation detail diagram. 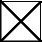 represents the dot product.

The detailed cross-attention calculation of layer1 process is shown in Figure 2.

The cross-attention operation of layer1 can be formulated by


(7) $$\begin{array}{l} O_{1}=\mathrm{softmax}\left(\frac{Q_{t}K_{a}^{T}}{\sqrt{d_{k}}}\right)\cdot V_{a}\\ O_{2}=\mathrm{softmax}\left(\frac{Q_{t}K_{p}^{T}}{\sqrt{d_{k}}}\right)\cdot V_{p} \end{array}$$

where $Q_{t}=\left\{qt^{1},qt^{2},\ldots ,qt^{n}\right\}$ is the query vector composed of time-domain feature sequences. $K_{a}=\left\{ka^{1},ka^{2},\ldots ,ka^{n}\right\}$ and $K_{p}=\left\{kp^{1},kp^{2},\ldots ,kp^{n}\right\}$ represent the key vectors composed of feature sequence in the frequency domain after linear mapping. Besides, $V_{a}=\left\{va^{1},va^{2},\ldots ,va^{n}\right\}$ and $V_{p}=\left\{vp^{1},vp^{2},\ldots ,vp^{n}\right\}$ are value vectors. ⋅ means the dot product of the matrix. $O_{1}$ and $O_{2}$ represent the output of the first layer of two cross-attention modules.

The cross-attention operation of next layer can be described as follows:


(8) $$O_{r}=\mathrm{softmax}\left(\frac{Q_{O_{1}}K_{O_{2}}^{T}}{\sqrt{d_{k}}}\right)\cdot V_{O_{2}}$$

where the query vector $Q_{O_{1}}$ is constructed by linear transformation of $O_{1}$ . $O_{2}$ is linearly transformed to obtain the key vector $K_{O_{2}}$ and the value vector $V_{O_{2}}$ . After that, $Q_{O_{1}}$ , $K_{O_{2}}$ and $V_{O_{2}}$ are fed into the next layer of the cross-attention module for deep feature fusion.

The result obtained after the cross-attention mechanism is the input of the FFN, which can be described as


(9) $$O_{FFN}=FFN\left(O_{r}\right)$$

where $O_{r}$ is the result of a two-level cross-attention module.

The output of the previous layer is subjected to an inverse convolution operation, which can be formulated as


(10) $$O_{\mathrm{final}}=\mathrm{softmax}(\mathrm{ConvTranspose}1D\left(O_{FFN}\right)$$

where $O_{FFN}$ is the output of FFN, and $O_{\mathrm{final}}$ is the identification result of CNNTF-*CA.* $\mathrm{ConvTranspose}1D\left(\cdot \right)$ represents the deconvolution operation, which performs up sampling on data to ensure that the output dimensions match the input dimensions.

## 4 Experiments and results analysis

### 4.1 Datasets

We select two signals, Binary phase Shift Keying (BPSK) and Quadrature Phase Shift Keying (QPSK), as the communication signal $S\left(t\right)$ . The carrier frequency is set to 2 MHz for signal $S\left(t\right)$ . In addition, the signal-noise-ratio (SNR) is set to [−20 dB, 18 dB] with an interval of 2 dB for the experiments in this paper.

For the interference data set, this paper firstly simulates five single interference signals, generates 1,000 samples under each SNR, each sample is sampled 1,024 times in the time domain. The parameters such as the center frequency, period and bandwidth of each type of interference signal are randomly distributed to simulate the real environment. Then the time domain data is changed by FFT to obtain the amplitude spectrum and phase spectrum data.

Under each SNR, the time domain, amplitude spectrum and phase spectrum are used as the three characteristics of the signal to splice and construct the data sets. The main simulation parameters for each type of interference signal are shown in Table 1. The interference signals are generated in MATLAB and model training and testing using python.

**Table 1:** Interference signal simulation parameters.

**Interference type**	**Parameter**
ST	Center frequency point random
MT	The number of tones is 3 ~ 8
LS	The initial frequency is randomly distributed between 20 MHz and 200 MHz, and the sweep slope is 20 ~ 100THz/s
PBN	The occupied bandwidth is random between 10 and 100 MHz
NFM	The mean value of modulated noise is 0, the variances is 1, and the frequency modulation coefficient $K_{\mathrm{fm}}$ =0.4 ~ 2

### 4.2 Performance of the proposed CNNTF

To evaluate the performance of our proposed method, the CNNTF are compared with the state-of-the-art methods including CNN (O’Shea et al., 2016), LSTM (Rajendrans et al., 2018), ResNet (West and O’Shea, 2017), CLDNN (Zhang et al., 2018), and GRU (Hong et al., 2017) in this paper.

Table 2 shows the overall recognition accuracy of each model on different sources. The overall accuracy represents the average recognition accuracy of each model for various types of interference under each SNR.

**Table 2:** Overall accuracy (%) of different models.

**Modulation mode**	**Model**	**Accuracy**
BPSK	CNN	73.54%
	LSTM	71.37%
	ResNet	75.41%
	CLDNN	74.88%
	GRU	74.42%
	**CNNTF**	**77.31%**
QPSK	CNN	76.54%
	LSTM	75.97%
	ResNet	77.66%
	CLDNN	80.22%
	GRU	78.54%
	**CNNTF**	**81.39%**

The bold values are our own proposed methods.

**Figure 3:**
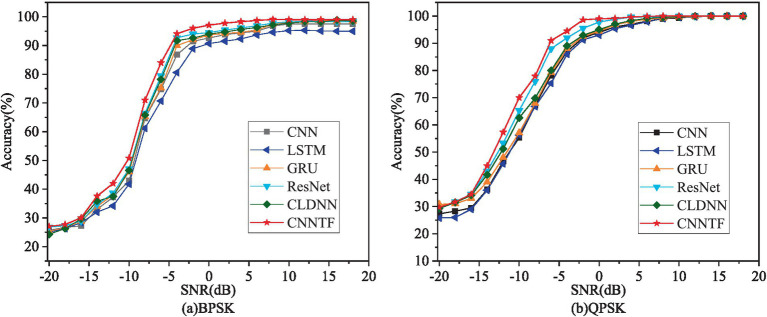
Accuracy diagram of interference recognition.

It can be observed that the method we proposed is higher in recognition accuracy than current mainstream methods. The average recognition accuracy of the six models for various types of recognition accuracy with SNR for six models is shown in Figure 3.

### 4.3 Performance of the proposed CNNTF-CA

Our proposed CNNTF demonstrates certain advantages over similar methods, owing to its capacity in extracting both global and local features, which brings in a high degree of information concentration. CNN, LSTM and GRU could not extract both global and local features. Compared with ResNet and CLDNN, which consider both global and local feature information, the advantages of the proposed CNNTF is slightly better. To further improve the performance, we introduced a cross-attention mechanism.

**Figure 4:**
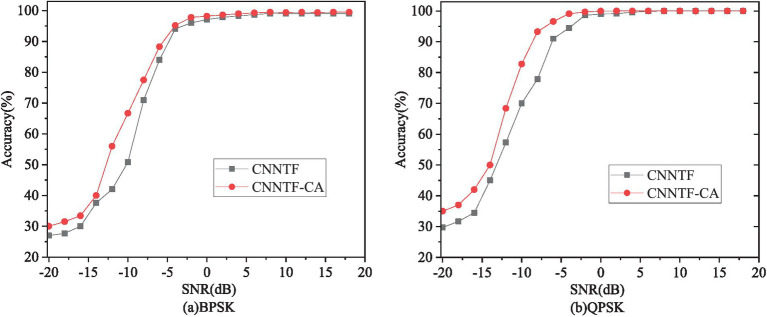
Overall recognition accuracy CNNTF and CNNTF-*CA.*

Figure 4 shows comparison chart of overall recognition accuracy between CNNTF and CNNTF-*CA.* From the figure, it can be observed that the recognition performance of CNNTF-CA has significantly improved under low SNR. The results are due to the use of the cross-attention mechanism, the time-frequency features are deeply correlated and the features are more differentiated between each type of modulated signal. CNNTF only performs simple feature splicing, so its performance is slightly worse than CNNTF-*CA.*

Table 3 presents the recognition performances of CNNTF-CA for each type of interference.

**Table 3:** Average accuracy (%) of CNNTF-*CA.*

Interference type	CNNTF-CA	
	BPSK	QPSK
ST	86.3%	90.1%
MT	81.0%	85.5%
LS	83.1%	85.3%
PBN	76.0%	81.8%
NFM	75.2%	82.5%

**Figure 5:**
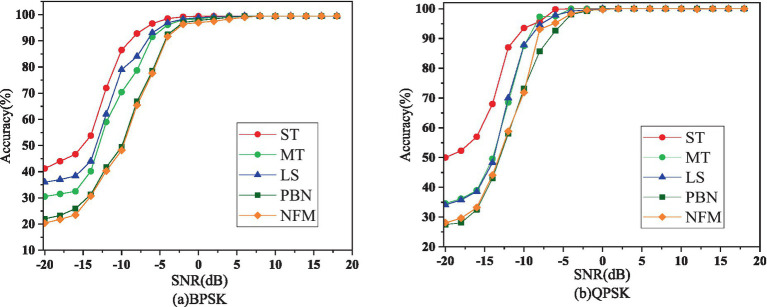
Identification accuracy of CNNTF-CA for each interference under BPSK and QPSK. Among them, ST is single-tone interference, MT is multi-tone interference, LS is linear scan interference, PBN and NFM represent partial band noise interference and noise frequency modulation interference respectively.

It can be seen from Table 3 that ST has the highest probability of being accurately identified among the five types of interference signals. In addition, the recognition effect of interference on QPSK is better than that on BPSK, which also shows that QPSK contains more information than BPSK. Simultaneously, PBN and NFM are the two types of interference that are most difficult to identify, whether under BPSK or QPSK. We display the recognition accuracy of CNNTF-CA for each interference in Figure 5.

The recognition accuracy of the CNNTF-CA approach for various interferences under BPSK and QPSK is depicted in Figure 5, as shown in this scientific figure.

It can be seen from the figure that the recognition accuracy curve of CNNTF-CA for different interferences has a similar trend, which also reflects the versatility and mobility of CNNTF-*CA.* We find that the recognition accuracy of different interference signals varies greatly, especially when the SNR is low.

**Figure 6:**
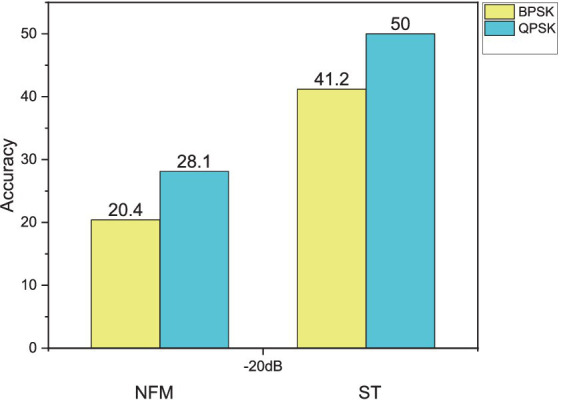
Identification accuracy of CNNTF-CA for each interference under BPSK and QPSK.

In order to present the results more intuitively, we use histograms in Figure 6 to depict the two signals with the best and worst effects in BPSK and QPSK at -20 dB, respectively. This approach aims to provide a more intuitive description of the results.

It is apparent that the model favors the identification of ST signals; however, its performance in recognizing NFM interference signals remains inadequate.

## 5 Conclusion

**Figure 7:**
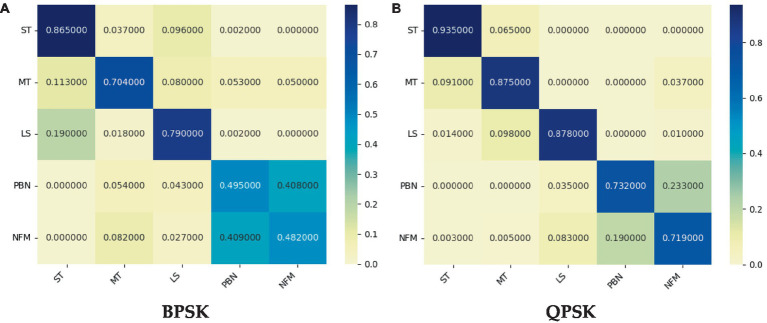
Confusion matrix of CNNTF-CA at -10 dB.

The performance of the proposed CNNTF-CA model is evaluated through the confusion matrices presented in Figures 7A,B for BPSK and QPSK, respectively, at a signal-to-noise ratio of -10 dB.

According to the confusion matrix illustrated in Figure 7, which represents the accuracy of identifying various interference signals under an SNR of -10 dB, it is apparent that the NFM and PBN signals exhibit relatively higher rates of misidentification when compared to the other signals present in the single interference data set. Specifically, the network demonstrates significant recognition errors in identifying NFM and PBN signals, highlighting a limitation that requires further attention in future research endeavors.

In addition, more precise assessment metrics can be derived based on Table 4. It can be seen that ST and LS are more likely to be correctly identified whether under BPSK or QPSK.

Furthermore, it is evident that regardless of the type of interference signal, the accurate recognition rate for QPSK is higher than that for BPSK, indicating the richer signal information contained within QPSK. These findings help the proposed model identify different interference signals faster and more accurately, playing a more important role in actual confrontation scenarios.

**Table 4:** Performance evaluation of CNNTF-CA under –10 dB.

**Types**	**BPSK**			**QPSK**		
	**Precision**	**Recall**	**F1-score**	**Precision**	**Recall**	**F1-score**
ST	0.741	0.865	0.798	0.896	0.935	0.915
MT	0.787	0.704	0.743	0.839	0.875	0.857
LS	0.763	0.790	0.776	0.961	0.878	0.918
PBN	0.475	0.495	0.485	0.794	0.732	0.762
NFM	0.513	0.482	0.497	0.720	0.719	0.719

In this paper, we propose a novel method that combines these CNN and transformer (CNNTF), to address the problem of identifying five single interferences. Given the challenge of extracting contextual semantics from one-dimensional signals using word encoding, this paper introduces a pioneering approach that exploits CNN instead. This novel combination, tailored to the unique data characteristics of one-dimensional signals, represents a significant contribution to the field. To further enhance the performance of the CNNTF model, we also incorporate a cross-attention mechanism that facilitates the correlation of the time and frequency domains of the input signals. This mechanism replaces the traditional approach of separate time-frequency analysis, leading to improved accuracy and efficiency in the identification and classification of different interference types. The effectiveness of the proposed approach is evaluated through extensive experiments and comparisons with other state-of-the-art methods. The experimental results demonstrate that the proposed CNNTF model with cross-attention mechanism achieves better performance in identifying and classifying different types of interferences.

Despite the promising results, it is important to acknowledge certain limitations and directions for future research. Current research is mainly limited to the evaluation of the CNNTF-CA model in simple scenarios. Further research on its performance under complex interference scenarios would be beneficial. To bridge the gap between theory and practical implementation, future research efforts will focus on optimizing the model’s robustness to changes in real-world signal conditions and extending its applicability to different signal interference environments.

## Data availability statement

The original contributions presented in the study are included in the article/supplementary material, further inquiries can be directed to the corresponding author.

## Author contributions

HZ: Conceptualization, Methodology, Writing – original draft. MeZ: Conceptualization, Methodology, Writing – original draft. MiZ: Funding acquisition, Writing – review & editing. SL: Writing – original draft, Writing – review & editing. YD: Writing – review & editing. HW: Supervision, Writing – review & editing.
